# The proteomic landscape of sperm surface deciphers its maturational and functional aspects in buffalo

**DOI:** 10.3389/fphys.2024.1413817

**Published:** 2024-06-28

**Authors:** Vipul Batra, Komal Dagar, Maharana Pratap Diwakar, Arumugam Kumaresan, Rakesh Kumar, Tirtha Kumar Datta

**Affiliations:** ^1^ School of Medicine, University of Nottingham, Nottingham, United Kingdom; ^2^ Animal Genomics Lab, ICAR-National Dairy Research Institute, Karnal, India; ^3^ Cell Science and Molecular Biology Lab, Animal Biotechnology Centre, ICAR-National Dairy Research Institute, Karnal, India; ^4^ Southern Regional Station of ICAR-National Dairy Research Institute, Karnal, India; ^5^ ICAR-Central Institute for Research on Buffaloes, Hisar, India

**Keywords:** buffalo, sperm, male fertility, shotgun-proteomics, reproduction

## Abstract

Buffalo is a dominant dairy animal in many agriculture-based economies. However, the poor reproductive efficiency (low conception rate) of the buffalo bulls constrains the realization of its full production potential. This in turn leads to economic and welfare issues, especially for the marginal farmers in such economies. The mammalian sperm surface proteins have been implicated in the regulation of survival and function of the spermatozoa in the female reproductive tract (FRT). Nonetheless, the lack of specific studies on buffalo sperm surface makes it difficult for researchers to explore and investigate the role of these proteins in the regulation of mechanisms associated with sperm protection, survival, and function. This study aimed to generate a buffalo sperm surface-specific proteomic fingerprint (LC-MS/MS) and to predict the functional roles of the identified proteins. The three treatments used to remove sperm surface protein viz. Elevated salt, phosphoinositide phospholipase C (PI-PLC) and *in vitro* capacitation led to the identification of N = 1,695 proteins (≥1 high-quality peptide-spectrum matches (PSMs), *p* < 0.05, and FDR<0.01). Almost half of these proteins (N = 873) were found to be involved in crucial processes relevant in the context of male fertility, e.g., spermatogenesis, sperm maturation and protection in the FRT, and gamete interaction or fertilization, amongst others. The extensive sperm-surface proteomic repertoire discovered in this study is unparalleled vis-à-vis the depth of identification of reproduction-specific cell-surface proteins and can provide a potential framework for further studies on the functional aspects of buffalo spermatozoa.

## 1 Introduction

The mammalian sperm surface is radically modified in its biomolecular composition and structure during its transit through the male and female reproductive tracts (MRT, FRT). A sperm’s journey in the FRT is arduous and tactical where they must bind reversibly with numerous epithelial surfaces simultaneously circumventing the phagocytes, antibodies and complement proteins (Bianchi and Wright, 2016; Archana et al., 2019; Voisin et al., 2019; Katila, 2012). The fertilizing spermatozoa must acquire surface properties primarily customized for this difficult voyage in the FRT. The transit through the MRT and capacitation in the FRT drastically remodel the testicular sperm surface which endows these spermatozoa with motility and competence for fertilization (Dacheux et al., 2009; Ikawa et al., 2010; Bianchi and Wright, 2016). For instance, numerous proteins in the immature testicular gametes are modified, removed, adsorbed or inserted either covalently or non-covalently (transiently or permanently) on their surface during their transit, particularly in the epididymis (Tecle and Gagneux, 2015; Batra et al., 2019). It has been posited that proteomic analysis is better suited to advance our understanding of sperm biology compared to genomic and transcriptomic analyses. This is because the gene expression varies vis-à-vis the cellular contexts and the transcribed mRNAs are not always translated. Besides, the proteins reflect the events downstream of gene expression and are considered to predict the phenotype of the cell more accurately vis-à-vis DNA and RNA (Kovac et al., 2013; Maverakis et al., 2015). Expectedly, the sperm surface too harbors a repertoire of heterogeneous proteins, which are not only responsible for their phenotype but also for their protection, survival, and function in the FRT (Cannarella et al., 2020; Binsila et al., 2021). This acquired sperm-specific proteome serves as the primary interface between the sperm and the various physiologic and immunologic milieus besides the surveillance mechanisms in the FRT (Dacheux et al., 2009; Tecle and Gagneux, 2015; Archana et al., 2019; Batra et al., 2020).

Buffalo is the major milk-producing animal in many countries, however, the poor reproductive efficiency of bulls (low conception rate after artificial insemination) constrains the realization of its full production potential (Minervino et al., 2020; Paul et al., 2021). Regardless of its economic significance in agriculture-based economies, the proteomic profile of buffalo sperm surface has not been investigated so far. Although a handful of studies are available on buffalo sperm proteome, these contemporary studies exhibit an inclination towards either seminal plasma proteins or biomarker discovery (Selvaraju et al., 2015; Codognoto et al., 2018; Fu et al., 2019; Hou et al., 2019; Ma et al., 2019; Karanwal et al., 2023). The sperm surface proteins mediate and facilitate numerous molecular and cellular interactions in various contexts including immunological, physiological and reproductive. Despite that, the buffalo sperm-surface proteome has largely been ignored which makes it difficult for researchers to explore and investigate the role of sperm proteins in the regulation of mechanisms associated with sperm protection, survival and function. Moreover, since the fertility of thousands of buffaloes is at stake by a single bull, it is therefore imperative to elucidate the buffalo sperm-surface proteomic profile and understand its molecular functions vis-à-vis sperm biology and male fertility.

Given the importance of sperm surface proteins in epididymal maturation, inter-cellular recognition, communication, capacitation, and fertilization in the FRT, an in-depth quantitative proteomic profile of buffalo sperm surface is required. This will not only indicate the nature of the interaction of these proteins, and their reproduction-specific functions but can also be referred to for the discovery of fertility-specific proteomic signatures. We have previously reported a partial proteome of buffalo spermatozoa wherein we demonstrated the existence of proteins implicated in reproduction and immune-related processes on buffalo spermatozoa (Batra et al., 2021). However, the study lacked the criteria for evaluating repeatability in the LC-MS/MS and appropriate controls. Consequently, to improve the depth of identification, we undertook this study to elucidate the proteomic repertoire of the buffalo sperm surface and predict the functional roles of the identified proteins associated with the buffalo sperm peripheral coats.

## 2 Materials and methods

### 2.1 Chemicals and plastic ware

All chemicals, media and reagents were procured from Sigma Aldrich Chemical Co. Ltd., (United States) unless stated otherwise. All plastic ware was procured from Nunc Inc. (Thermo Scientific, United States). Fetal bovine serum (FBS) was obtained from Hyclone, Canada.

### 2.2 Semen sample collection and pre-processing

Neat semen samples (mass motility ≥3) were collected from Murrah buffalo bulls of proven fertility (*N* = 12 bulls, aged between 3–5 years) using an artificial vagina at the Artificial Breeding Research Centre (NDRI, Karnal, India). The ejaculates were collected in 15 mL Falcon centrifuge tubes containing Sp-TALP i.e., HEPES buffered Tyrode’s medium (pH 7.4, 38°C) referred to as non-capacitating medium (NCM). The semen was transported to the laboratory at 38°C and was washed thrice by centrifugation at 700 *g* for 10 min to separate the seminal plasma components. The motile spermatozoa were subsequently selected by subjecting the final pellet to the swim-up technique and thereafter the motile sperm fraction was resuspended in variable volume (200–800 μL) of the NCM according to the downstream experiments.

### 2.3 The removal of buffalo sperm-surface proteins

We used three different strategies for removing surface proteins from the motile spermatozoa fraction. The motile buffalo sperm were subjected either to elevated salt extraction (2X-DPBS) or 2 U/mL Phosphatidylinositol-specific phospholipase-C (PI-PLC) incubation or *in vitro* capacitation to remove the non-covalently (electrostatic interactions) bound, GPI-anchored, capacitation induced proteins, respectively. The elevated salt extraction and PI-PLC extractions of buffalo sperm-surface proteins were done as per the procedure described by Batra and colleagues (Batra et al., 2021). Briefly, the post-swim-up spermatozoa were incubated in 2X-DPBS (elevated NaCl) for 30 min in siliconized micro-centrifuge tubes at 38°C with gentle shaking and subsequently pelletized by centrifugation at 280 *g* for 10 min. For the elevated salt protein removal method, the spermatozoa incubated in 1X-DPBS were considered control. The supernatants were pooled, filtered through a 0.22 μm filter and frozen at −80°C until further use. For PI-PLC incubation, 100 × 10^6^ spermatozoa were incubated with PI-PLC (2 U/mL) from *Bacillus cereus* (Sigma) in non-capacitating Sp-TALP medium (NCM) for 2 h with gentle shaking at RT. Subsequently, the samples were centrifuged at 1,000 *g* for 10 min and the supernatants were pooled and filtered through a 0.22 μm filter and frozen. The PI-PLC treatment control sample was processed similarly, however, was devoid of the PI-PLC enzyme. It is now widely accepted that the majority of the epididymal and seminal plasma proteins (SPPs), including the decapacitation factors must be shed from the sperm surface to allow capacitation and fertilization (Hernández-Silva et al., 2020). Amongst these, we have previously reported the removal of BuBD-129 from the buffalo sperm surface upon exposure to elevated salt and PI-PLC (Batra et al., 2021). However, the effect of capacitation on its interaction with the buffalo sperm surface is unknown. We extracted the sperm surface proteins released during capacitation by inducing *in vitro* capacitation of buffalo spermatozoa, as described previously (Batra et al., 2020). Briefly, 400 × 10^6^ spermatozoa were suspended in the capacitating medium (NCM supplemented with 6 mg/mL bovine serum albumin (BSA), 2 mM CaCl_2_.2H_2_O and 10 mM NaHCO_3_) for 6 h in a 5% CO_2_ incubator at 38°C after sequentially washing in the capacitating medium (CM) containing 1 and 3 mg/mL BSA. The spermatozoa incubated in NCM-only for 6 h were considered control for the *in vitro* capacitation. After the incubation period, the samples were centrifuged at 600 *g* for 10 min and the supernatants from four ejaculates were pooled and filtered. The sperm capacitation was confirmed by chlortetracycline (CTC) staining, as mentioned below. Only the samples containing more than 50% of capacitated spermatozoa were used for further experimentation. The proteins in the filtrate obtained from the three treatments were precipitated using the acetone-precipitation method (1:9, supernatant/acetone ratio), concentrated on a SpeedVac vacuum concentrator and subjected to SDS-PAGE after quantification by Quick Start™ Bradford protein assay.

### 2.4 Assessment of sperm function parameters

After protein removal, the control and protein-removed buffalo spermatozoa were evaluated for intactness of structural and functional integrity and to confirm the occurrence of capacitation. The assessment of sperm-membrane integrity was done by using carboxyfluorescein diacetate and propidium iodide (CFDA-PI), as per the method described by Singh and colleagues (Singh et al., 2016). The assessment of mitochondrial membrane potential (MMP) by JC-1 (5,5′,6,6′-tetrachloro-1,1′,3,3′-tetraethylbenzimidazolyl-carbocyanine iodide) and capacitation status by Chlortetracycline (CTC), a fluorescent cheated probe of Ca^2+^ was done by the methods described by Saraf and co-workers (Saraf et al., 2019). After staining, the excess stains were removed by washing the stain-incubated spermatozoa with 200 µL of sperm-TALP by centrifugation at 800 *g* for 3 min. The pelleted spermatozoa were used to make a thin smear onto which a few drops of mounting medium, Dabco^®^ 33-LV were placed and observed at 1,000X magnification under an Olympus BX-51 fluorescence microscope (fitted with a digital CCD, DP71 camera) using either the blue (excitation wavelength 470–490 nm, emission wavelength 510 nm) or UV (excitation wavelength 355–375 nm, emission 420 nm) or green filter (excitation wavelength 520–550 nm, emission 580 nm) or combinations thereof. A minimum of *n* = 250 spermatozoa (in triplicates) were evaluated, in a minimum of 10 fields for observing fluorescent patterns. The images of the two filters were merged to obtain the final image, wherever required.

### 2.5 Electrospray ionization, tandem mass spectrometry (LC-MS/MS) analysis

The acetone precipitated protein from the three extraction treatments were initially dissolved in 6 M guanidium hydrochloride. Subsequently, 25 μL of the dissolved samples were reduced with 5 mM TCEP and then alkylated with 50 mM iodoacetamide (in the dark). Next, the samples were digested with trypsin (1:50, trypsin/lysate ratio) for 16 h at 37°C (in-solution digestion). Afterwards, the digests were cleaned using a C18 silica cartridge and dried using a SpeedVac vacuum concentrator. The dried pellet was resuspended in Buffer A (5% acetonitrile, 0.1% formic acid). All the experiments were performed using an EASY-nLC 1,000 system (Thermo Fisher Scientific) coupled to a Thermo Fisher-*QExactive* mass spectrometer equipped with a nano-electrospray ion source. The peptide mixture (1 μg) was resolved using a 25 cm PicoFrit column (360 μm outer diameter, 75 μm inner diameter, 10 μm tip) filled with 1.8 μm of C18-resin (DrMaeisch, Germany). The peptides were loaded with Buffer A and eluted with a 0%–40% gradient of Buffer B (95% acetonitrile, 0.1% formic acid) at a flow rate of 300 nL/min for 100 min. The electron spray ionization (ESI) was the source of ions, and the MS scan mode was Orbitrap. The collision-induced dissociation (CID) was the mode of fragmentation (y and b ions) and the linear ion trap was the MS/MS scan mode (400–2000 m/z). The MS1 resolution was set to 70,000 while the MS2 resolution was 17,500. The MS data were acquired using a data-dependent top10 (most intense peaks) method that dynamically selects the most abundant precursor ions from the survey scan. To check the performance of the instrument, a 20 fM standard BSA digest was parallel analyzed. The mass spectrometry proteomics data have been deposited to the ProteomeXchange Consortium *via* the PRIDE (Perez-Riverol et al., 2019) partner repository with the dataset identifier PXD028026.

### 2.6 Data processing and analysis

After sample processing, the generated RAW files were analyzed with Proteome Discoverer (*ThermoFusion* v2.2) against the NCBI buffalo reference proteome database translated from the latest chromosome level genome assembly (*UOA_WB_1*). For the Sequest search, the precursor and fragment mass tolerances were set at 10 ppm and 0.5 Da, respectively. The protease used to generate peptides, *i.e.*, enzyme specificity was set for trypsin/P (cleavage at the C terminus of “K/R: unless followed by “P”) along with a maximum missed cleavage value of two. Carbamidomethyl on cysteine as fixed modification and oxidation of methionine and N-terminal acetylation were considered as variable modifications for database search. The peptide-spectrum match and protein false discovery rates (estimated by searching the data against a decoy database) were set to 1% to ensure only high-confidence protein identifications for the peptide-spectrum match (PSM) and site decoy fraction. The proteins identified with a minimum of one unique peptide (≥1 high-quality PSM per peptide) were used for analysis. Some of these peptides are likely legit and could be missed if the “minimum two peptides” criterion was used. Therefore, the MS/MS spectra of these proteins were also manually verified. The downstream statistical analysis was performed in the Perseus environment (Tyanova et al., 2016) wherein the LFQ-intensity (abundance values from PD v.2.0) values were log_2_ transformed. The data was imputed for missing values by assuming normal distribution using the implicit algorithm. The quantitative differences in the LFQ-intensity values of extracted proteins after treatments were analyzed by the two-tailed *t*-test (assuming equal variance within the groups of replicates) with Benjamini–Hochberg false discovery rate (FDR)-correction of the *p*-value (correction of the alpha error) at<0.01 significance level. The volcano plots were used to analyze two-tailed significance values identified by determining fold change vs. –log_10_ fold change. A *p*-value<0.05 and FDR≤0.01 were considered statistically significant.

### 2.7 Validation of identified protein (β-defensin-129) in the mass spectrometric (LC-MS/MS) analysis

We have previously reported that BuBD-129 was the maximally expressed β-defensin in the buffalo male reproductive tract (Batra et al., 2019). Its functional ortholog in macaques is reportedly released from the sperm surface upon induction of capacitation (Yudin et al., 2003). We selected BuBD-129 for Western blot (WB) assays to validate and evaluate the effect of *in vitro* capacitation on its interaction with sperm surface. The sperm proteins were extracted as described previously (Karanwal et al. 2023). Briefly, the control and capacitated samples were centrifuged at 600 *g* for 10 min and the sperm pellet was re-suspended in 500 μL RIPA buffer supplemented with Halt protease inhibitor cocktail. The samples were agitated on an orbital shaker at 4°C overnight to ensure complete lysis of the spermatozoa. Next day, the supernatants were collected by centrifugation at 16,000 × g for 30 min at 4°C. The extracted proteins in the supernatant were quantified by Quick Start™ Bradford protein assay, as per the manufacturer’s protocol to determine their concentration. The absorbance of the standards and the unknown samples was read at 595 nm on Infinite^®^ 200 NanoQuant microplate reader (Tecan). The extracted protein (15 μg) samples were subjected to discontinuous SDS-PAGE (12% resolving and 5% stacking gels) analysis followed by transfer onto a Polyvinylidene-fluoride (PVDF) membrane using a mini-VE vertical electrophoresis system (Hoefer). The PVDF membranes (blots) were incubated either with mouse monoclonal anti-β-tubulin antibody (T8328, Sigma-Aldrich, 1:5000) or custom synthesized polyclonal antibody specific for BuBD-129 (1:75,000) at 4°C overnight with slight agitation (Batra et al., 2021). After probing with a primary antibody, the blots were washed thrice in TBST for 10 min. Later, the blot was probed with the optimized dilution of HRP conjugated secondary antibody for 2 h at RT: HRP-labeled anti-rat IgG (1:20,000 or 1:80,000 dilution; AP136P, Sigma Aldrich). The membranes were washed 4 times in TBST with each washing for 15 min before detecting the bound antibodies. The immunoreactivity was detected by using chemiluminescence (Luminata forte substrate, Merck), as per the manufacturer’s instructions. The immunoreaction signals were captured using CL-XPosure Film (Thermo Scientific).

### 2.8 Functional annotation of the identified proteins

The proteome for water buffalo (*Bubalus bubalis*) was not available at the time of proteomic data analysis for this manuscript. Besides, most of the gene/protein annotation software, gene ontology and functional annotation tools do not list buffalo as a reference organism. Therefore, we used the NCBI buffalo database reference proteome database (UOA_WB_1) and the translated coding sequence file, translated CDS (.faa) as the search database (and decoy). The sequence-derived functional information was generated through BLAST2GO 5.0, a platform for high-quality functional annotation and analysis of high-throughput omics datasets (Conesa et al., 2005). It transfers the existing functional annotations from existing annotated sequences to the uncharacterized novel sequences (e.g., for Buffalo). It uses functional attributes of gene ontology (GO) analysis to assess the biological meaning of the mined data, which was subsequently visualized using MS Excel. For the annotation and identification of enriched pathways, the functional enrichment analysis web tool, KOBAS 3.0 was used. It is a KEGG orthology-based annotation system (KOBAS), used for automated annotation and pathway identification of novel and uncharacterized proteins. It annotates a given set of genes with potential pathways and disease associations by mapping them to genes with known annotations which allows both ID mapping and cross-species sequence similarity mapping. The pie donut diagrams were generated using HighCharts. Additionally, a thorough survey of available literature (published after 2010) on PubMed was performed for functional annotation of identified proteins using the keywords semen, sperm, testis and epididymis. The article results from both human and animal models were included and no criteria on design, sample size, or methodological standards were placed. All the duplicates were removed and titles that lacked mention of the considered keywords were excluded.

## 3 Results

The macroscopic and microscopic parameters of semen were analyzed and evaluated before any downstream experiments. The ejaculates that were either milky or creamy in colour, 2–4 mL in volume, free from coagulation, and homogenous in their consistency were considered for processing. The samples were subsequently observed microscopically for normal morphology. The ejaculates with a mean sperm concentration of more than 600 × 10^6^/mL were considered for the swim-up procedure.

### 3.1 Protein extraction treatments hamper functional integrity in a subset of buffalo sperm

**FIGURE 1:**
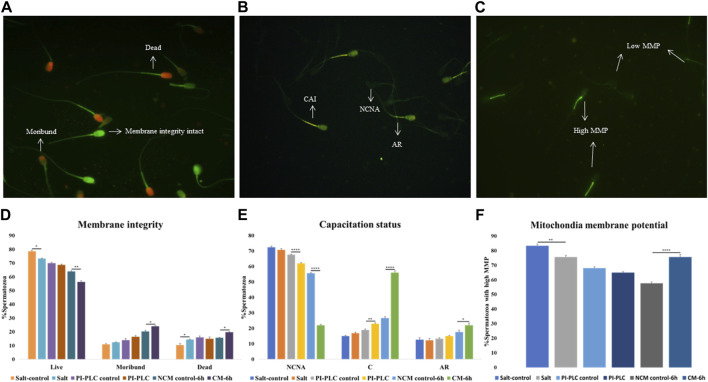
Sperm Functional Parameters (SFPs) The buffalo spermatozoa were subject to three surface protein extraction treatments viz. Elevated salt extraction (2X-DPBS) or 2 U/mL Phosphatidylinositol-specific phospholipase-C (PI-PLC) treatment or *in vitro* capacitation and the functional parameters were assessed along with the respective controls (see text). CFDA with PI (see text) was used to assess the membrane integrity of the spermatozoa that were categorized as live, dead or moribund **(A, D)** CTC (see text) was used to categorize the spermatozoa as non-capacitated (NC), capacitated **(C)**, or acrosome-reacted (AR) **(B, E)** JC-1 (see text) dye was used to assess the mitochondrial membrane potential (MMP) and the spermatozoa were distinguished as with high or low MMP **(C, F)**. Different letters denote statistical difference (*p* < 0.05), *p < 0.05, **p < 0.01, ***p < 0.001, ****p < 0.0001.

The CFDA-PI dual staining distinguished three spermatozoa populations viz. live (membrane integrity intact), dead (membrane integrity impaired) and moribund (dying sperm, source of ROS) spermatozoa based on their fluorescence patterns indicative of the functional or compromised membrane integrity (Figure 1A). The plasmalemma integrity of the buffalo spermatozoa appeared to be compromised resulting in a significant decline in the percentage of sperm with intact plasma membrane integrity (live) upon exposure to elevated salt (*p* < 0.05) and induction of *in vitro* capacitation (*p* < 0.01) compared to their respective controls (Figure 1D).

Capacitation is a crucial process that allows sperm cells to mature and attain the fertilizing ability in the FRT. We used Chlortetracycline (CTC) to confirm the occurrence of capacitation in the buffalo sperm after the stipulated period of *in vitro* induction of capacitation (6 h). The fluorescence microscopy determined three fluorescent patterns classified as non-capacitated (NCNA), capacitated (CAI) and acrosome-reacted (AR) spermatozoa (Figure 1B). Surprisingly the exposure of buffalo sperm to PI-PLC increased the percentage of capacitated sperm (*p* < 0.01) while the induction of *in vitro* capacitation resulted in a significant rise in the percentage of spontaneous acrosome reaction (*p* < 0.05) and capacitated spermatozoa (*p* < 0.0001), as expected (Figure 1E).

The mitochondrial membrane potential (MMP) is a potent marker of sperm health. The JC-1 dye was used to assess the MMP of buffalo sperm after protein removal procedures. This dye provides bright green fluorescence to the spermatozoa with high MMP whilst the spermatozoa with low MMP produced a dim green-ish fluorescence after labelling with JC-1 exhibiting the expected. The fluorescence is preferentially localized to the sperm mid-piece that harbors the mitochondria (Figure 1C). The mitochondrial membrane potential (MMP) of buffalo spermatozoa diminished upon exposure to elevated salt causing a significant reduction (*p* < 0.01) in the population of sperm mitochondria bearing high MMP (Figure 1F). Contrarily the percentage of buffalo spermatozoa bearing high MMP elevated significantly (*p* < 0.0001) upon induction of *in vitro* capacitation.

Overall, the exposure of buffalo sperm to elevated salt appeared to perturb the plasmalemma integrity and MMP while the exposure to PI-PLC induced premature capacitation in a subset of buffalo spermatozoa.

### 3.2 Buffalo sperm surface harbours thousands of distinct proteins

**FIGURE 2:**
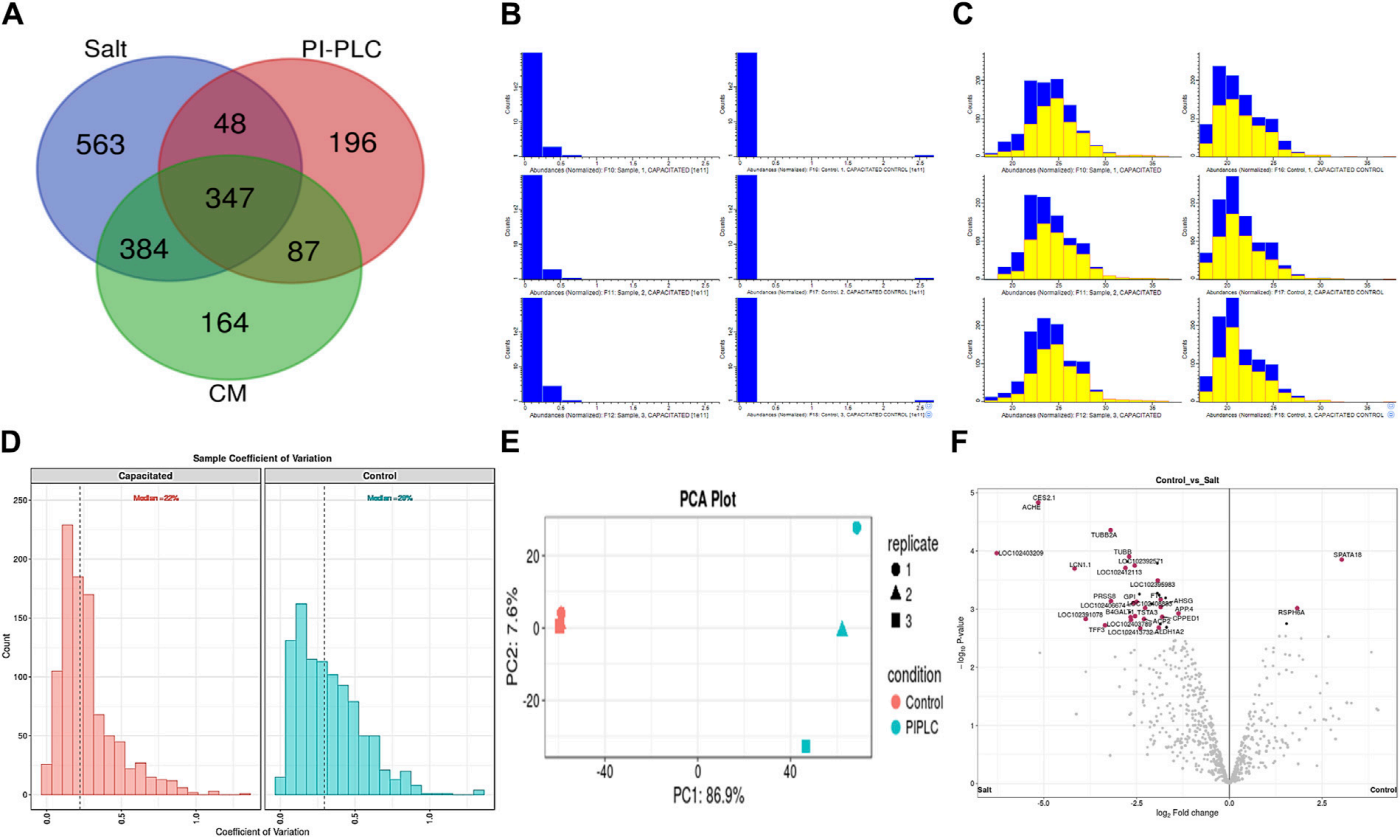
The sperm surface proteins, data pre-processing, statistical analysis, and cross-sample and replicate comparisons. Proteome Discoverer (v2.2) identified 1,342, 678, and 982 distinct proteins and isoforms (*p* < 0.05, FDR<0.01) in the salt-extracted, PI-PLC treated and capacitated samples, respectively **(A)**. The Perseus software platform (v2.0.2.0) was used for normalizing the sample protein abundances and interpreting their quantification. Representative histograms depict the normalized abundances before transformation **(B)** and after transformation **(C)** with significantly abundant proteins marked in yellow. The computation of sample coefficient of variation to understand the sample variability **(D)** and dimensionality reduction using principal component analysis, PCA **(E)** were also performed in Perseus v2.0.2.0. The volcano plots were used to assess the statistical significance (-log10 *p*-value) vis-à-vis the magnitude of change (log_2_ Fold change) for the differential abundance proteins across the three samples and their controls, e.g., elevated salt and its control **(F)**.

A total of 64,290, 46,526 and 33,902 PSMs corresponding to 4,881, 1781 and 2,782 unique peptides, respectively were identified from buffalo sperm surface after extraction by elevated salt (2X-DPBS), PI-PLC and *in vitro* capacitation. These PSMs and peptides were mapped to 1,342, 678 and 982 proteins (*p* < 0.05, and FDR<0.01) were identified in the three samples, respectively as illustrated (Figure 2A; Supplementary Sheet-Results). Overall, we report a cumulative identification of 1,695 proteins from the three protein removal methods employed in this study.

**FIGURE 3:**
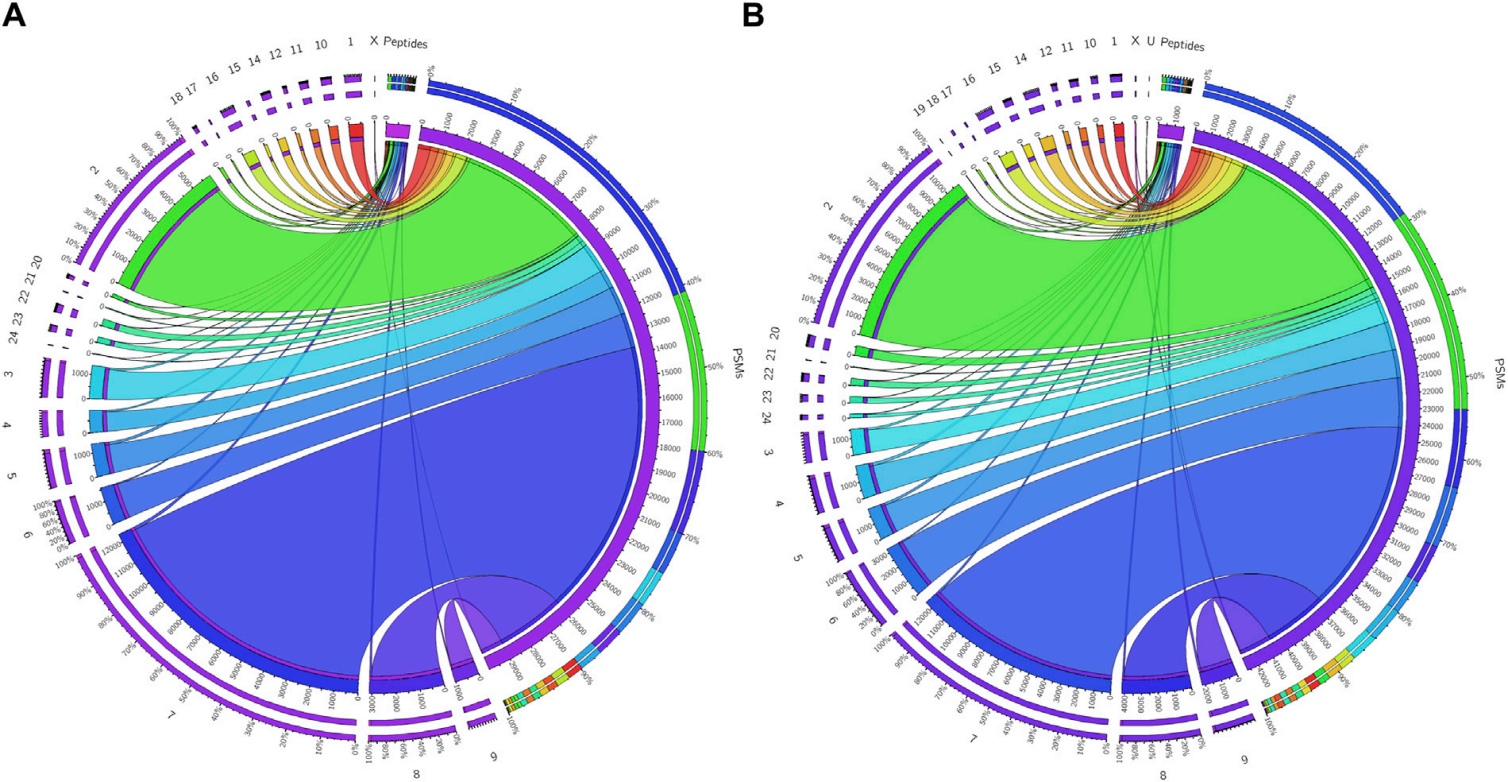
Chromosome mapping of buffalo sperm-surface proteins Circos plots (chord plots) rendered as ratio layouts indicating the individual chromosomal localization of the identified peptides which revealed an uneven distribution of chromosomal protein expression, e.g., in before **(A)** and after **(B)** PI-PLC treatment of buffalo spermatozoa. Ribbons in the ratio layouts represent the relationship between the number of peptide-spectrum matches (PSMs) and the number identified peptides coded from each of the depicted chromosomes. Thickness of the ribbon indicates the number of PSMs/identified peptides (proteins) from that chromosome.

**FIGURE 4:**
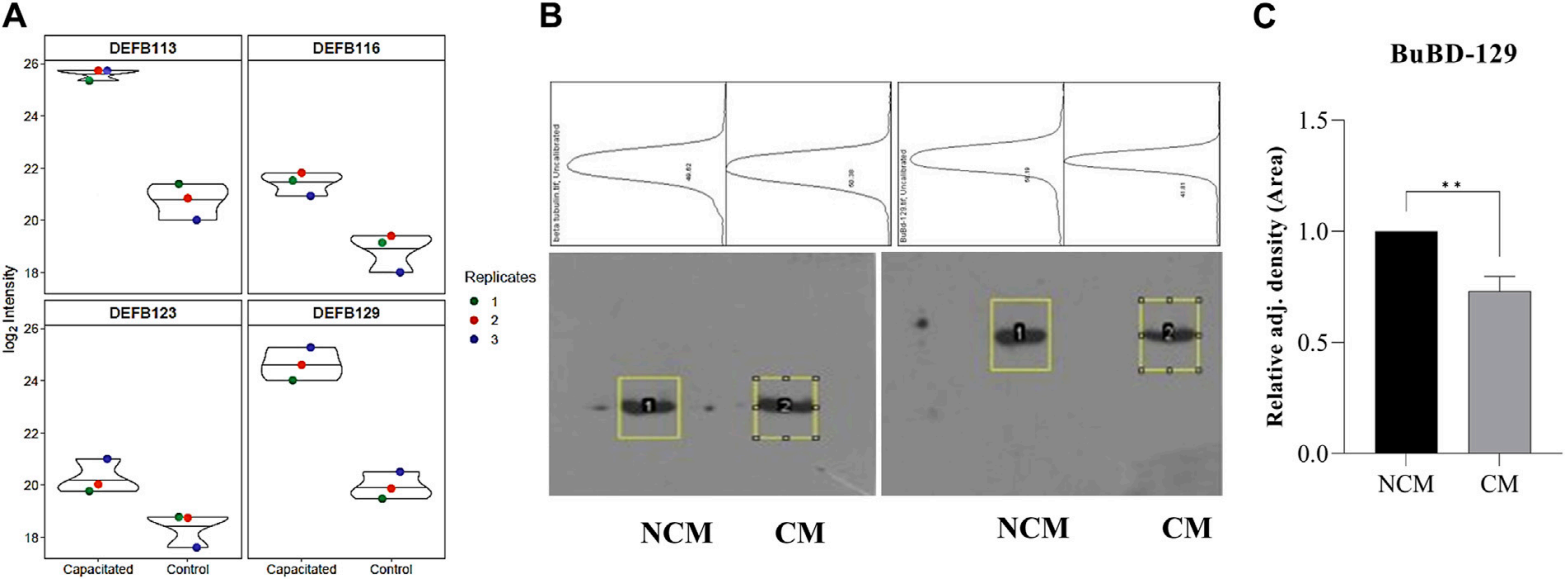
Overrepresented proteins The violin plots for overrepresented proteins beta-defensins (DEFBs) were computed for the assessment of the log2 normalized abundance values across the sample and technical replicates for all three treatment groups, e.g., capacitation **(A)**. Blot images showing relative abundance of beta-tubulin and BuBD-129 in non-capacitated (NCM), control and capacitated (CM) spermatozoa **(B)**. The in-house generated polyclonal anti-BuBD-129 produced a prominent band at ∼35–40 kDa in both control and CM sperm (**B**, right). The mouse monoclonal anti-β-tubulin antibody (T8328, Sigma-Aldrich) produced a band at 50 kDa (**B**, left) and served as the reference control. Densitometry analysis was done in ImageJ and the adjusted density values indicated the removal of BuBD-129 after induction of *in vitro* capacitation **(C)**. **p < 0.01.

The initial screening of the buffalo sperm-surface proteins indicated the highly diversified nature of these proteins vis-à-vis physical and chemical parameters. The identified proteins and their isoforms exhibited a wide linear dynamic range in abundance, identity, molecular mass, number of amino acids and pI indicating the differential effects of the used protein-removal methods (Supplementary Sheet-Results and Supplementary Figure S1). For example, the isoelectric point for XP_025127145.1 was predicted to be 4.06 whereas the highest pI in the sample was predicted for XP_006071250.1 as 11.87, a difference of seven orders of magnitude (Supplementary Sheet-Results). Likewise, the lowest and highest molecular weight was predicted to be 7.8 and 781.4 kDa for XP_025148992.1 and XP_025129505.1 a difference of nearly 10 times! The Perseus software platform (*v*. 2.0.1.0) was used for interpreting the protein identification and quantification, normalization, cross-replicate comparisons (and establishing the correlation between replicates from similar or distinct samples) and multi-hypothesis testing for the multi-dimensional proteomics data generated in this study (Figures 2B–F; Supplementary Figure S2A). Surprisingly only 25 proteins were differentially abundant (*p* < 0.05) in elevated salt (2X-DPBS) treatment in comparison to its control (1X-DPBS) whilst 197 and 623 proteins (*p* < 0.05) were found to be differentially abundant in PI-PLC exposed and *in vitro* capacitated samples vis-à-vis their respective controls. We also sought to determine the individual chromosomal localization of the identified peptides by creating Circos plots (chord plots). Although the contribution from all the chromosomes was observed, the distribution of protein expression was uneven. Notably, the removal of the GPI-linked proteins by PI-PLC significantly changes the mapping dynamics albeit with maximum peptides mapped on chromosome 7 (Figure 3; Supplementary Figure S3A, B). These differentially abundant proteins (DAPs) were further considered for downstream enrichment and pathway analysis. Interestingly, amongst the identified DAPs, numerous beta-defensins (host defense peptides, HDPs) were found to be among the overrepresented and the differential abundance of beta-defensins BuBD-113, 116, 123 and 129 were deemed statistically significant (*p* < 0.05) in the capacitated sample vis-à-vis control sample (Figure 4A). We validated the differential abundance of BuBD-129 in control and capacitated sperm samples using Western blot, which indicated its removal (*p* < 0.01) upon the induction of *in vitro* capacitation in buffalo sperm (Figures 4B, C). Interestingly, BuBD-129 was the only beta-defensin common in all three protein-removal procedures employed in this study.

### 3.3 Functional annotation of the identified proteins elucidates the reproduction-specific sperm surface proteome

**FIGURE 5:**
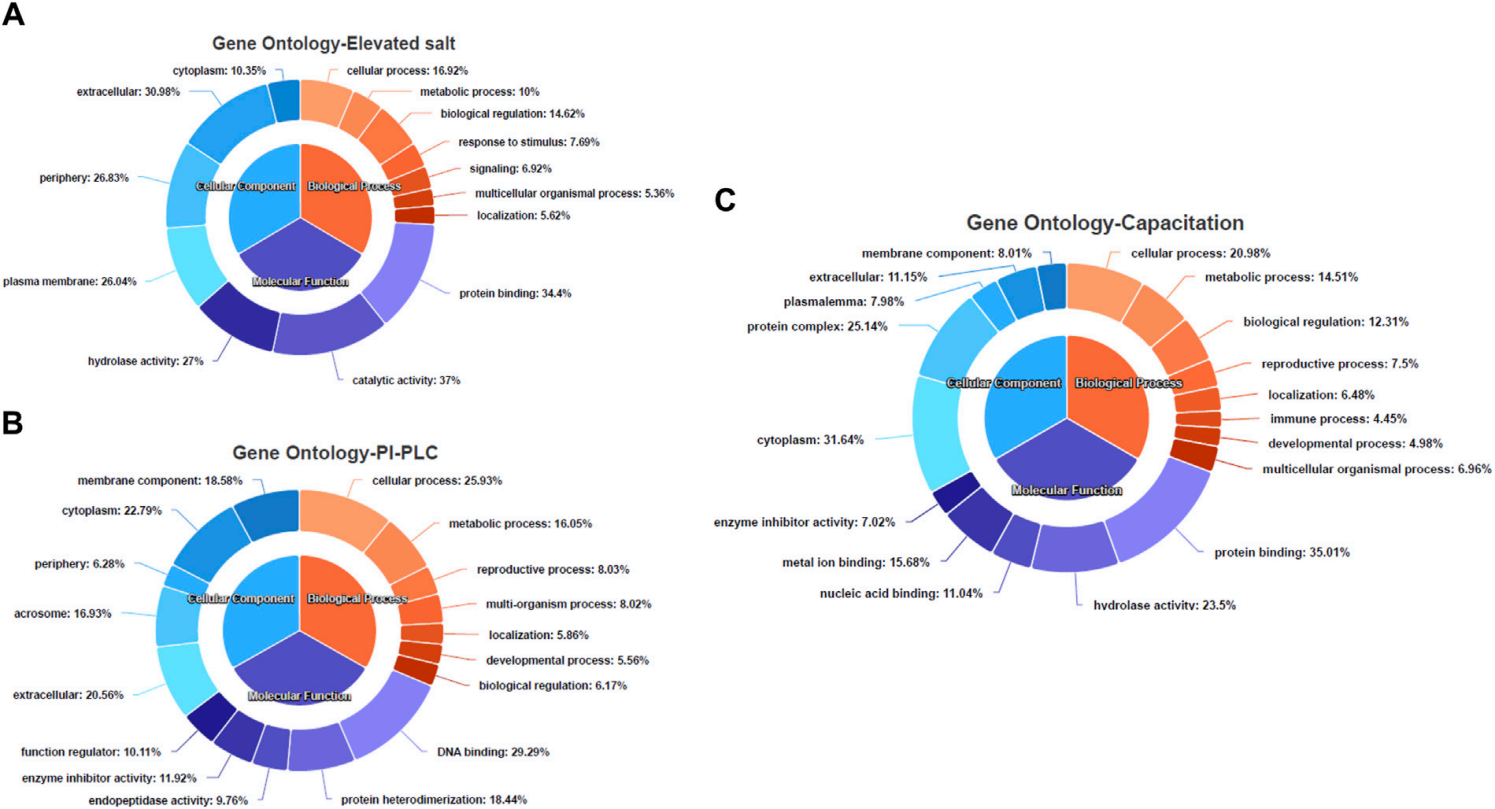
The functional annotation of the LC-MS/MS identified proteins Pie-donut charts indicating the results of BLAST2GO analyses illustrating biological process, molecular function and cellular component ontology terms across the elevated salt **(A)**, PI-PLC treated **(B)** and capacitated spermatozoa **(C)**. The GO IDs from every significant alignment for each sequence were directly annotated to the sequence if the alignments similarity passed the desired minimum and validated to remove the intermediate GO terms.

The gene ontology (GO) and pathway analyses for the 25, 197 and 623 DAPs were carried out to hint at the biological processes and molecular functions associated with buffalo sperm surface proteins extracted by elevated salt (2X-DPBS), PI-PLC and *in vitro* capacitation. The BLAST2GO 5.0 uses the pair-wise similarity search algorithm allowing a deduction of specific functions played by the identified proteins by extrapolation of function from sequence orthologs (Consea et al., 2005). The maximum number of hits were returned from *B. bubalis* (buffalo) followed by *Bos taurus*, cross-bred cattle and other ruminants (Supplementary Figure S3C–E). The overall frequency distribution of the GO-level functional annotation of the biological process, molecular function and cellular component terms of the gene ontology analysis indicating the annotation quality is depicted in Supplementary Figure S4A–C. While most of the identified proteins across the three treatments were extracellular or peripheral (*p* < 1.0E-6) (Figure 5A–C), the PI-PLC treatment released proteins localized to the acrosomal vesicle (Figure 5B). Contrarily, the capacitation led to the release of a considerable amount (31.64%) of the intracellular proteins (Figure 5C). Surprisingly, the identified protein across the three treatments exhibited a considerable overlap in the functional categories of the GO terms (*p* < 1.0E-6) and were involved in metabolic and cellular biological processes apart from enrichment in processes involved in reproduction, development and biological regulation, nevertheless, with differential number of genes dedicated to these processes (Figure 5A–C). Besides, the functional categories (*p* < 1.0E-6) associated with the immune system and reproduction-associated processes were found to be enriched for proteins isolated by *in vitro* capacitation (Figure 5C). While most of the proteins released by salt treatment were predicted to possess catalytic (37%) or enzymatic (hydrolase, 27%) activity, only a fraction (9.76%) of the GPI-APs were predicted to possess enzymatic activity (endopeptidase). Nevertheless, nearly one-fourth of the capacitation-related proteins (23.4%) were predicted to possess hydrolase activity and a high and exclusive presence of the metal-ion/protein binding domain (Figure 5C). Amongst the proteins identified in the PI-PLC and elevated salt treatments, 22.79% and 10.35%, respectively were predicted to be intracellular (cytoplasm).

**FIGURE 6:**
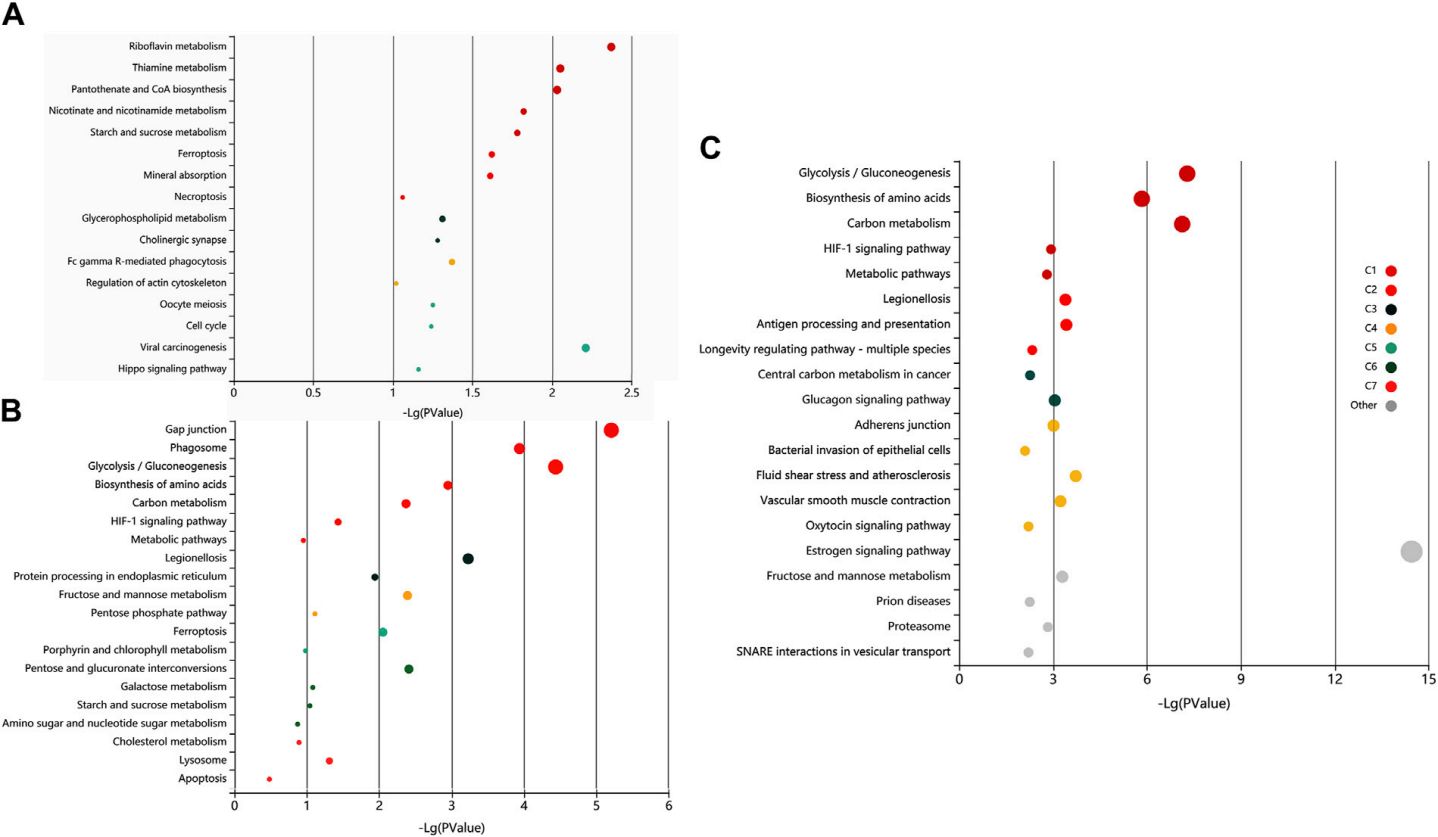
Exploratory visualization of the enriched terms from pathway analysis by KEGG orthology-based annotation system (KOBAS) KOBAS 3.0, was employed for automated annotation and pathway identification of novel and uncharacterized proteins which revealed a significant enrichment (P-valued<0.05, FDR<0.01) of metabolic, homeostasis and response to the extracellular signal, and signaling and immune-related pathways in elevated salt **(A)**, PI-PLC treated **(B)** and capacitated spermatozoa **(C)**. Bubbles represent *p*-values for pathway terms in different clusters consisting of colour mega-cluster (from C1 to C7) and top clusters not matched to any mega-cluster (light grey bubbles). Interrelated pathways are clustered and rendered in the same color.

The pathway analysis of the identified proteins was done using KOBAS 3.0. It is a web server for annotating and identifying the enriched pathways in novel sequences (sequence-based) based on mapping to genes with known annotations. The data-set analysis revealed significant enrichment (P-valued<0.05, FDR<0.01) of metabolic, homeostasis and response to the extracellular signal, and signalling and immune-related pathways in the non-covalently bound (2X-DPBS), GPI-anchored and protein released upon induction of *in vitro* capacitation (Figure 6).

**FIGURE 7:**
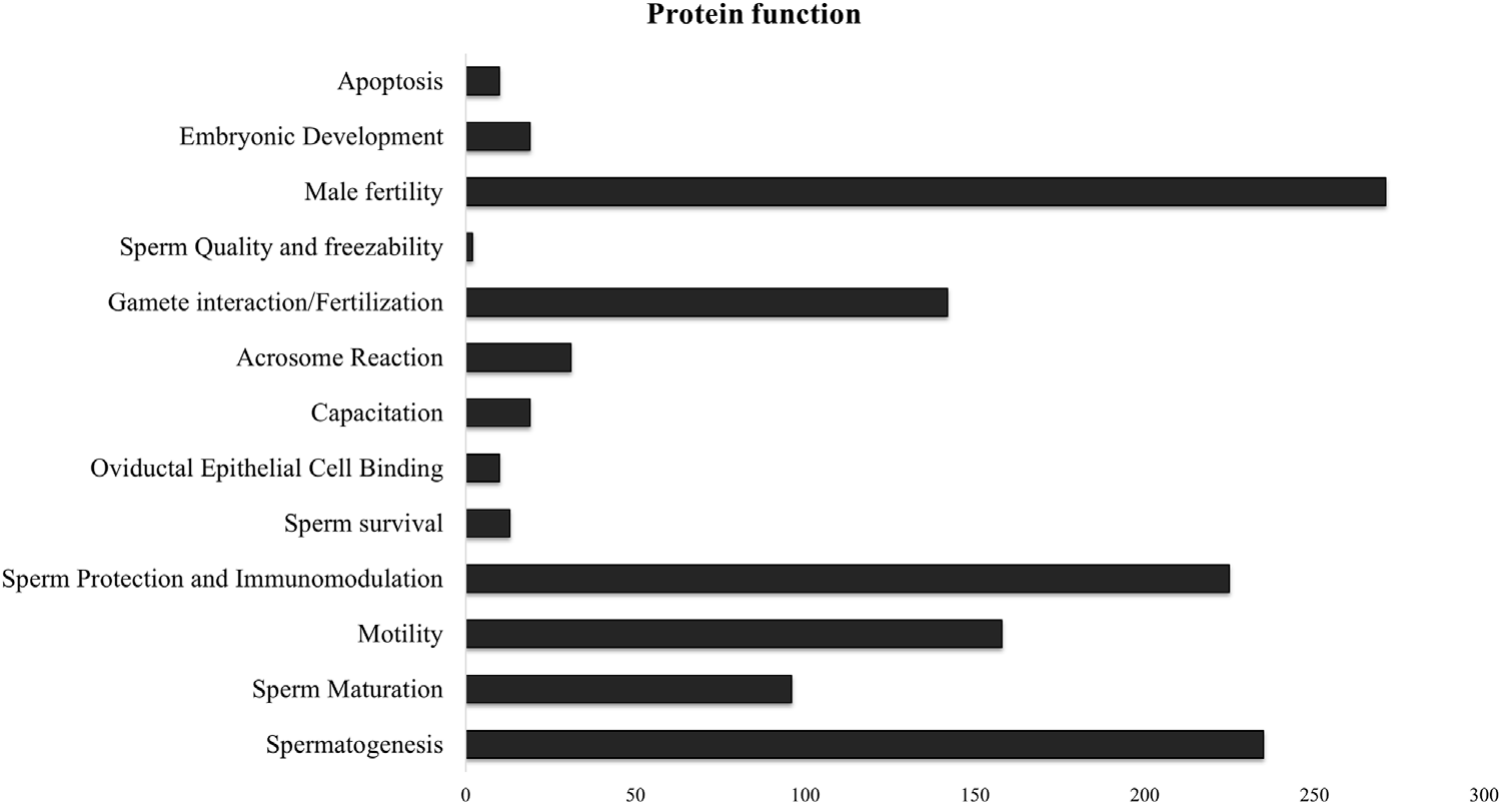
Sperm surface-specific functional proteomic repertoire The literature-based functional characterization of buffalo sperm surface-specific *N* = 873 unique protein isoforms identified across the samples indicated their involvement in the regulation of various aspects of male reproductive physiology mainly sperm production, survival and function. Most of the identified proteins are implicated in spermatogenesis, sperm protection and regulation of male fertility.

**FIGURE 8:**
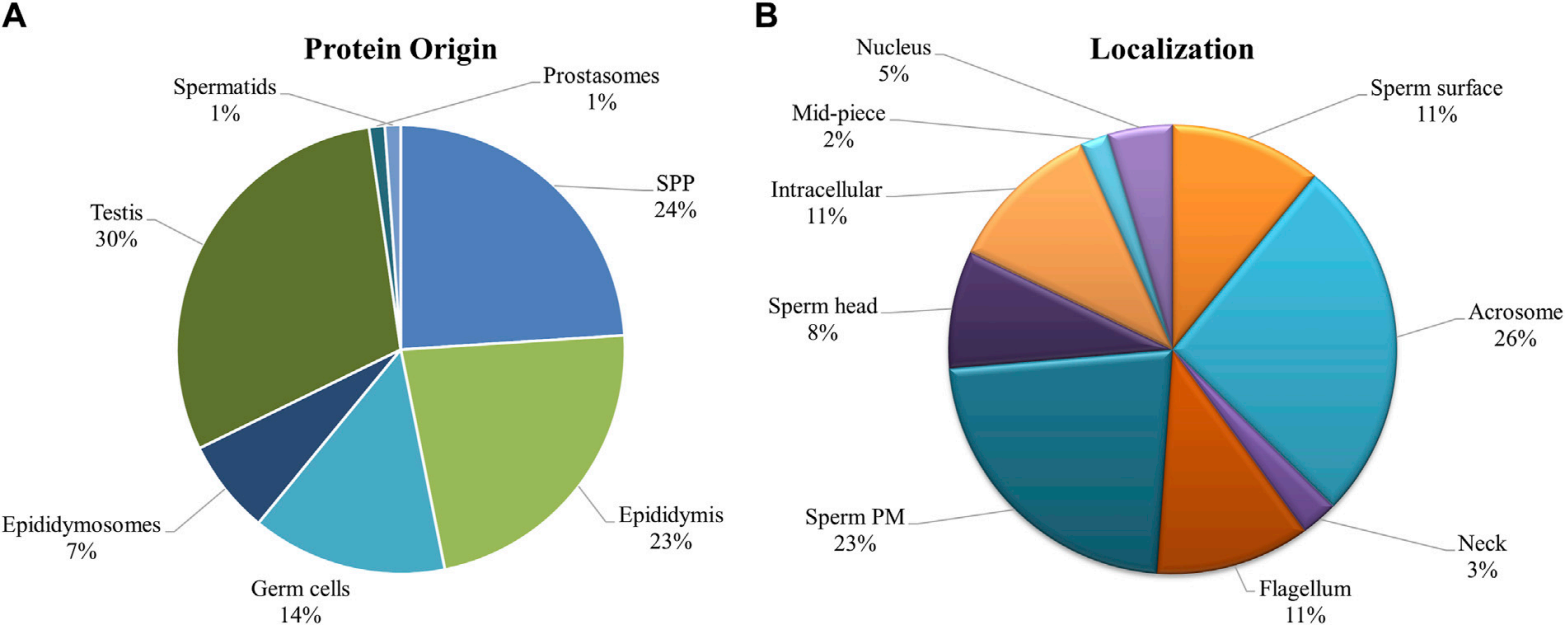
Origin and Localization. Pie charts indicating the source or the origin **(A)** and sub-cellular location **(B)** of the buffalo sperm surface-specific 873 unique proteoforms identified across the samples, as inferred from the literature search. Almost a third of the proteins originate in testis and another third are added in the epididymis (and epididymosomes). Most of the proteins were predicted to be on the sperm exterior. SPP, Seminal Plasma Protein, PM, Plasma Membrane.

Since there was significant overlap and similarity in the functional categories of the GO terms, we performed a literature search for reproduction-specific functional annotation of identified proteins. Our extensive literature search for each of the identified proteins (*N* = 1,695) revealed that more than 50% of the proteins (N = 873) were implicated in male reproductive physiology context. The analysis revealed that most of the proteins were involved in reproduction-specific processes, e.g., regulation of male fertility, spermatogenesis and sperm protection followed by motility, gamete interaction and sperm maturation (Figure 7). The maximum numbers of proteins were found to be testicular in origin followed by almost an equal contribution from the epididymis and seminal plasma amongst others (Figure 8A). A majority of these proteins (∼60%) were reportedly localized to sperm periphery including acrosome, plasma membrane or sperm surface (Figure 8B).

## 4 Discussion

We presented a straightforward methodology for discovering the various populations of proteins that interact with the buffalo sperm surface and play crucial roles in sperm survival, protection and function. The identification of *N* = 1,342, 678, and 982 distinct proteins in the salt-extracted, PI-PLC treated, and capacitated samples, respectively, not only highlights differences in protein interaction with buffalo sperm surface but also demonstrates varying effects of extraction treatments in removing non-covalently attached, GPI-anchored, and capacitation-associated proteins. Our study provides a cumulative repertoire of *N* = 1,695 sperm surface proteins that will be valuable to decipher the maturational and functional aspects of sperm biology. The overall GO and pathway functional annotation analysis revealed a rich proteomic background covering a myriad of functions. As expected, there was an enrichment of proteins (*N* = 873) involved in the regulation of fertility, spermatogenesis, maturation, protection (immunomodulation) and zona-binding or penetration. Most of the identified proteins were relevant in the context of male fertility and thus provide a platform for further studies on sperm biology.

We have previously reported the removal of sperm surface protein by elevated salt and PI-PLC treatments (Batra et al., 2021). Despite using multiple biological replicates, stringent search criteria and statistical rigour, our previous study had limitations in terms of lesser technical replicates and a shorter MS run (60 min). Therefore, to increase the identification depth and confidence, we used *N* = 12 biological replicates, and *n* = 3 technical replicates for each sample group besides increasing the length of the MS/MS run to 100 min. Additionally, we included the proteins released at the time of in vitro-induced capacitation. The shotgun proteomics (LC-MS/MS) approach used in this allowed us to determine the effect of extraction treatments and helped to attain an unparalleled depth of *N* = 1,695 buffalo-specific proteoforms. This study offers greater depth compared to previous proteomic analyses of buffalo sperm. Earlier methods, such as 2D gel electrophoresis coupled to MS, provided valuable insights into fertility-related proteins but were limited by technical constraints, e.g., examining only a subset of proteins (Rahman et al., 2013; Somashekar et al., 2015; Archana et al., 2019; Ma et al., 2019). The protein inventory list generated in this work will shed light on the functional roles of peripheral and transmembrane proteins hitherto unknown to exist on the buffalo sperm surface. We employed BLAST2GO 5.0 (OmicsBox) and KEGG Orthology Based Annotation System (KOBAS 3.0) for functional and pathway annotation of the identified proteins considering the lack of high throughput, annotated bio-molecular data for buffalo (Bu et al., 2021). The GO term cellular component indicated the enrichment of proteins localized to the plasma membrane, cell-periphery, and acrosome vesicle or secreted in the extracellular space (Figure 5). This indicated the successful extraction of sperm-surface, plasma membrane proteins by the method used in this study. The identified proteins were predicted to be primarily involved in the regulation of metabolism, the immune system (immunomodulation) and reproductive processes (e.g., gamete interaction) and most of them possessed one or other catalytic domains or enzymatic activity (Figures 5A–C, 7). These observations agree with the earlier studies presenting the evidence of expression of genes in spermatozoa that are crucial to their functional attributes (Kawano et al., 2012; Sendler et al., 2013; Selvaraju et al., 2017).

One limitation of this study is the identification of some intracellular proteins among the surface-specific proteins. However, as expected, few sperm cells had compromised membrane integrity while others were precociously capacitated (Figure 1). It would unavoidably lead to the release of intracellular proteins in the incubation medium. This is because the sub-cellular compartments of mammalian spermatozoa are known to be highly dynamic architectures which are sensitive to extraction treatments (López-Salguero et al., 2020). The identification of additional, non-GPI-anchored proteins after the PI-PLC treatment is another limitation of this study. It has been reported that many of the non-covalently bound proteins to the GPI-anchored proteins ought to be released along with them upon PI-PLC exposure (Yudin et al., 2002). This along with the proteins released during untimely sperm maturation may explain identification of acrosome proteins in the PI-PLC treated sample and the occurrence of common and intracellular proteins between the three treatments. It however remains unclear, which of the identified intracellular proteins are true contaminants or the ones that exist and function on the buffalo sperm surface. Many of these proteins hitherto thought to be intracellular have now been identified on sperm surface, e.g., HSPA2, HSP60, PGK2, AK1 (Asquith et al., 2004; Kongmanas et al., 2015). Furthermore, it has been reported that many of the core intracellular proteins, e.g., nucleosomal histones (H1, H2A, H2B, H3 and H4) can directly translocate across the cellular membrane and act as carriers for the import of extracellular macromolecules into living mammalian cells (Hariton-Gazal et al., 2003). This may be true for some or many of such proteins identified in this study, however, this needs to be validated. The following section discusses the major functional classes of proteins identified in this study:

The origin and development of the haploid sperm from the germ cells starts with the mitotic division of spermatogonial stem cells in the seminiferous tubules of the testis (Staub and Johnson, 2018). We also identified proteins implicated in spermatogenesis including DAZAP1, HNRNPK, HNRNPC and HNRNPM in all three sperm extraction treatments. The Deleted in Azoospermia Associated Protein 1 (DAZAP1), which is predominantly a nuclear protein in late-stage spermatocytes and round spermatids has been demonstrated to localize to the cytoplasm in elongated spermatids (Lin et al., 2006). The heterogeneous nuclear ribonucleoproteins C1/C2 and M (HNRNPC, HNRNPM) are known to co-localize with DAZAP1 in the nucleus and have been implicated in mRNA transport during spermatogenesis (Lin et al., 2006). Other nuclear ribonucleoproteins such as sumoylated hnRNP2AB1 have also been found to be responsible for sorting miRNAs into EVs by selective sorting of small RNAs in activated T-lymphocytes (Villarroya-Beltri et al., 2013). Similar mechanisms involving the RNA binding proteins may assist the selective sorting of small RNAs into epididymosomes and explain their presence on the sperm surface.

Unlike most spermatogenesis events, the concluding phases of the differentiation of spermatozoa occurring in the epididymis do not appear to be under the genomic control of the germline. This is because, as the differentiation progresses in the testes, the DNA starts condensing. Consequently, the transcription in the germline DNA eventually diminishes and then ultimately halts (Dacheux et al., 2003; Gatti et al., 2004; Cornwall, 2014). Subtle interactions between the sperm and the components in the luminal milieu of the epididymis modify the surface of spermatozoa in a series of sequential biochemical modifications. Many of these components bind transiently, for example, molecules acquired in the distal epididymal regions, which are obligatory to traverse the array of mucosal fluids and extracellular matrices in the FRT (Diekman, 2003; Robaire and Hinton, 2015). We also identified several proteins involved in sperm maturation and capacitation. Many of these proteins are involved in modifying the sperm plasma membrane which affects their function and survival in the FRT. For example, Lipocalins, which are secretory proteins, involved in the transport and storage of secondary metabolites and several family members have been identified in epididymal fluid and on sperm surface (Guyonnet et al., 2009; Hamil et al., 2003). Contrarily, other epididymal proteins affect the epididymal sperm maturation indirectly, e.g., low-density lipoprotein receptor-related protein 8 (LRP8) also known as apolipoprotein (apo) E receptor-2 (apoER2). It is a member of the low-density lipoprotein receptor family which is a cell surface receptor implicated in ligand endocytosis and signal transduction (Myant, 2010) We also identified a common ligand of LRP8, the apolipoprotein E (ApoE) which plays a crucial role in phospholipid and cholesterol homeostasis. Upon interacting with LRP8, ApoE is taken up by cells and may remain intracellular or move to the cell surface, while LRP8 is cleaved by gamma-secretase (Reddy et al., 2011). It has been proposed that ApoE is the signaling ligand for ApoER2 in regulating the JNK signaling pathway (Reddy et al., 2011). Interestingly, ApoER2 also interacts with C-jun-amino-terminal kinase-interacting proteins 1 and 2 (JIP1 and JIP2) which are scaffold proteins that regulate stress-activated MAPK (mitogen-activated protein kinase)) signaling. Interactions with varied ligands including JIPs and ApoE indicate that LRP8 forms a scaffold for various interactors at the cell surface (Stockinger et al., 2000; Whitmarsh, 2006). LRP8 protein is highly expressed in caput epididymis and has been reported to influence the functional expression of clusterin and phospholipid hydroperoxide glutathione peroxidase (PHGPx) resulting in abnormal sperm morphology and immotility (Keber et al., 2013; Andersen et al., 2003). Clusterin (CLU) is a pleiotropic extracellular chaperone glycoprotein synthesized and secreted by the testis, epididymis, and seminal vesicle. It is mainly found on the surface of abnormal spermatozoa and is associated with reduced motility, sperm aggregation, elevated DNA fragmentation and strong morphological alterations (Ibrahim et al., 2000; Schröter et al., 1999; Muciaccia et al., 2012). However, in humans, rodents, and sheep it is also found on the motile, mature and morphologically normal sperm (Sylvester et al., 1991; Ibrahim et al., 2001a; Ibrahim et al., 2001b; Janiszewska and Kratz, 2020). The secretory isoform of clusterin, sCLU is present in semen, however, the epididymal proteoform is localized over the entire sperm head in the inner plasma membrane of normal human spermatozoa (Han et al., 2012; Kongmanas et al., 2015; Saewu et al., 2017). In bulls, the fraction of CLU positive spermatozoa (CPS) in an ejaculate have been proposed as a reliable indicator of male fertility than sperm motility or abnormal morphology. This is because the percentage of the CPS are in a significant inverse relationship with the estimated relative conception rate, a very accurate method for determining fertility (Ibrahim et al., 2000). Interestingly, CLU is also known as apolipoprotein J and reportedly binds to LRP8 (ApoER2) which triggers a Reelin-like signal resulting in the activation of PI3K/Akt (Leeb et al., 2014). Notably, the localization pattern of sperm CLU is altered upon capacitation translocating from entire sperm head to the head-hook region in mice (Saewu et al., 2017). Accordingly, we observed a higher abundance of CLU in elevated salt and PI-PLC treatments vis-a-vis capacitation (Supplementary Sheet-Results). These capacitation-associated changes in sperm clusterin localization patterns have also been proposed as potential biomarkers of motility, capacitation, and ejaculate quality (Saewu et al., 2017; Kumar et al., 2012). Reportedly, CLU also plays crucial roles in sperm maturation and survival in the FRT. For instance, CLU can inhibit complement induced sperm lysis thus protecting the sperm from membrane attack complex (MAC) (Janiszewska and Kratz, 2020; Long, 2020). In humans, CLU along with the cysteine-rich protein EPPIN (Epididymal protease inhibitor) and lactotransferrin (LTF) forms a protein complex (EPC) on the surface of epididymal spermatozoa (Wang et al., 2007). Eppin (also called SPINLW1; serine peptidase inhibitor-like with Kunitz and WAP domains 1) is localized to the head and the flagellum of the mature human sperm (Mariani et al., 2020). The protein complex comprising CLU (a metalloproteinase inhibitor), EPPIN and LTF (both microbicidal) provides protection to spermatozoa and may act as a sperm plasma membrane receptor indicating its key role in a network of protein-protein interactions on the sperm surface (Wang et al., 2007; Mariani et al., 2020). Expectedly, we detected the three EPC components, nonetheless, the fourth member of the EPC, semenogelin (SEMG1) or its ortholog Seminal-vesicle secreted protein 2 (SVS2) was not detected (Gomes et al., 2023). This may be because the SEMG1 is a seminal plasma protein (SPP) (Wang et al., 2007) and we separated the seminal plasma components during initial semen processing. Overall, CLU is deemed crucial for various sperm functions including regulation of capacitation and maintaining immune tolerance for male antigens in the female reproductive tract. These results underpin the fact that innate immunity and sexual reproduction are entwined in an intricate relationship. We also report an abundance of immune-related proteins associated with the stabilization of the sperm membrane during the immune attack by immune cells and immunomodulation, particularly in the uterine lumen, as previously reported (Holland and Nixon, 1998; Kirchhoff, 1998; Légaré et al., 2017; Batra et al., 2021). For instance, we identified 11 unique β-defensins (DEFBs) in this study viz. DEFB-107A-like, 110, 112, 113, 114, 116, 119, 121, 123, 43-like and DEFB-129 (BuBD-129). Such a high abundance of these host-defence peptides (HDPs) agrees with previous "‘omics” studies that have also demonstrated a high expression of DEFB genes that are known to be implicated in evading the elicited immune responses (protection) and regulation of sperm function (Guyonnet et al., 2009; Batra et al., 2021). Many of the sperm transcripts of β-defensins such as DEFB-1, 7, 123, 124, 119 and DEFB-110 have been reported to be highly expressed in HF bulls and thus implicated in the regulation of male fertility (Binsila et al., 2021; Ma et al., 2019; Hou et al., 2019; Paul et al., 2021). Interestingly, BuBD-129 was the only β-defensin common to all three extraction methods. This novel defensin has been demonstrated to embellish the entire buffalo sperm and is predicted to be heavily O-glycosylated and a potential ortholog of primate DEFB-126 (Batra et al., 2019; Batra et al., 2021). This may explain the anomaly between the observed band at ∼35–40 kDa and the computed molecular weight (MW) which is ∼19 kDa. (Figure 4B). Similar effects of O-glycosylation have been reported to cause a shift in the observed molecular weight of beta-defensins (Tollner et al., 2004; Tollner et al., 2011; Tollner et al., 2012). DEFB-126 is a pleiotropic host defense peptide (HDP) that promotes the progressive motility of the corpus sperm, resulting in an increase in cervical mucus penetration ability (Fernandez-Fuertes et al., 2016; Tollner et al., 2008a), countering uterine immune responses (Yudin et al., 2005), formation of oviductal sperm reservoir (Tollner et al., 2008b; Lyons et al., 2018), capacitation (Yudin et al., 2003) and gamete interaction (Tollner et al., 2004). Its functional ortholog in buffaloes, BuBD-129 (DEFB129) was reported amongst the most expressed prominent sperm coat proteins (Batra et al., 2019; Batra et al., 2021), which could be a potential fertility biomarker like DEFB-126 (Tollner et al., 2004; Tollner et al., 2011; Tollner et al., 2012). Future studies on elucidating its exact physiological function would unravel its exact role in the modulation of buffalo bull fertility. The cysteine-rich defensin-like peptides (e.g., DEFBs) including the members of cysteine-rich secretory proteins, antigen 5 and pathogenesis-related 1 proteins (CAP) superfamily are integral to the reproductive success of organisms ranging from invertebrates and plants to higher primates (Avila et al., 2011; Okuda et al., 2009; Amien et al., 2010; Tollner, 2012). CRISP1, a member of the CAP protein superfamily is a cysteine-rich secretory protein (CRISP) implicated in regulation of sperm capacitation and is involved in sperm oocyte fusion (Roberts et al., 2006; Cohen et al., 2011). We consistently detected CRISP1, along with BuBD-129 (DEFB-129) across all the three extraction treatments. Both CRISP1 and DEFB-129 have been reported to be upregulated in the caput epididymis from men with non-obstructive azoospermia (Dubé et al., 2008). In mice, epididymal CRISP1 has been demonstrated to enhance the progressive motility of sperm under non-capacitating conditions (Hu et al., 2018). Besides, it functionally cooperates with CRISP4 to establish optimal acrosome function and sperm-egg interaction (Hu et al., 2018). It has now been established that CRISP1 can regulate the key calcium channel in sperm, CatSper which is crucial for hyperactivation, a vigorous motility essential for penetrating the egg’s vestments (Ernesto et al., 2015). Overall, CRISP proteins are crucial to sperm transport in the FRT, capacitation, gamete interaction and thus male fertility (Gonzalez et al., 2021). Besides DEFBs and CRISP proteins, other proteins implicated in sperm protection and immunomodulation, e.g., stress-responsive heat shock proteins (HSP90, HSPA1L, HSPA2, HSPB1, HSPA6, HSPA4, HSPA8, HSPB9), Cathelicidin-4 and 5, membrane cofactor protein CD-46, CD-5, CD-59, BOLA class I histocompatibility antigen and complement factor H were also observed. Many of these proteins reportedly regulate sperm-specific functions. These proteins also assist the mammalian sperm to improve their survival through various immunomodulatory, repair and protection mechanisms apart from their roles in sperm-specific biological functions such as capacitation, sperm-ZP interaction and fertilization (Naaby-Hansen and Herr, 2010; Bansal et al., 2015; Baena et al., 2018; Weber et al., 2020; Binsila et al., 2021). For example, the complement factor H identified in this study is a known complement regulator. It has two proteoforms and both the seminal plasma and acrosome proteoforms display similar complement regulatory activity and protect the mammalian sperm from complement-mediated damages in the MRT and FRT (Sakaue et al., 2010; González-Cadavid et al., 2014). Another complement regulator, the membrane cofactor protein (MCP) aka CD-46 was also identified in this study. This protein is expressed as four proteoforms out of which two proteoforms were found to be expressed in seminal plasma (ssMCP) and on spermatozoa (smMCP) (Seya et al., 1993). The smMCP is reportedly localized to the inner acrosomal membrane and has been reported to be critical for the survival of the acrosome-reacted spermatozoa by regulation of complement activation and the interaction of spermatozoa with oocytes in the FRT (Cervoni et al., 1992; Riley et al., 2002). Notably, the CD-46 sperm-specific proteoform, smMCP has distinct structural features of a lower molecular weight and modifications (deglycosylation) in the N-linked sugars (Riley, 2002). Nonetheless, other CD family members and other proteins, e.g., BuBD-129 (Batra et al., 2021) identified in this study are known to be highly glycosylated. CD59 (protectin), for example, is a GPI-anchored glycoprotein that has been identified across the plasma membrane of fresh ejaculated, capacitated and acrosome-reacted spermatozoa. CD59 along with CD46 (MCP) and decay accelerating factor (CD55) play a crucial role in protection against complement-mediated lysis and gamete interaction (Fenichel et al., 1994; Schröter et al., 1999). Besides, the abundance of immune-related proteins has also been associated with the stabilization of the sperm membrane during the immune attack by immune cells and immunomodulation, particularly in the uterine lumen (Légaré et al., 2017; Holland and Nixon, 1998; Kirchhoff, 1998). Overall, a considerable proportion of the identified proteins were implicated in sperm protection, and immunomodulation thus ensuring the survival of the fertilizing spermatozoon.

The sperm and the seminal plasma proteins (SPPs) play crucial roles in a variety of reproduction-specific biological functions and are hence implicated in the regulation of male fertility. For example, the amyloid beta-protein precursor (APP) superfamily members, many of which are known to be involved in cell survival and protection (Park et al., 2010). Nonetheless, they are expressed in tissues outside the nervous system, e.g., Alzheimer APP which is a testicular protein with a crucial role in sperm development (Shoji et al., 1990; Silva et al., 2015). It has been reported that the Alzheimer APP is detected only during spermatogenesis and was thus proposed to be implicated in cellular differentiation or morphologic change (Shoji et al., 1990). In a recent study, APPs were identified as pivotal proteins in male reproduction due to their diverse biological functions, including the regulation of male sexual behavior, and their extensive protein interaction networks within testis and spermatozoa (Park et al., 2010; Silva et al., 2015). It is worth mentioning that moonlighting enzymes such as carboxylesterase 4A (CES4A) and 5A (CES5A) were also identified in this study. These proteins are found across phyla and have been reported to exhibit redundancy and over-expression in the MRT (Mikhailov and Torrado, 2000). CES5A, mainly found in the corpus and cauda epididymides, inhibits capacitation, and reduces sperm motility in Ces5a-knockdown rats, resulting in decreased male fertility (Ru et al., 2015). Amongst the stress-response proteins identified in this study, the heat shock proteins (HSP) family members have now been identified to play crucial roles in the functioning of the mammalian sperm. For example, the HSP90 α and β are critical regulatory proteins that are expressed on the sperm surface and are important to progesterone-induced progressive motility and acrosome reaction (Ecroyd et al., 2003; Purandhar et al., 2014; Sagare-Patil et al., 2017; Ramesha et al., 2020). We noted a higher abundance of another family of sperm-surface proteins, the Hsp70 family and its members viz. heat shock 70 kDa, heat shock 70 kDa protein 1-like (HSP1AL), heat shock-related 70 kDa protein 2 (HSPA2), heat shock 70 kDa protein 4 (HSPA4), heat shock 70 kDa protein 4 L (HSPA4L), and heat shock 70 kDa protein 6 (HSPA6). The Hsp70 family members appear to be amongst the most abundant components of the sperm surface (Miller et al., 1992; Kamaruddin et al., 2004; Purandhar et al., 2014) and their altered expression, e.g., of Hsp70-2 could be associated with the pathogenesis of male infertility (Feng et al., 2001; Purandhar et al., 2014). HSPA2 is a testis-enriched proteoform of the HSP70 family that is considered a prime regulator of zona pellucida-receptor complex assembly (Redgrove et al., 2012). Many of the heat shock proteins, e.g., HSPA8, HSPA1B, HSPA1L, HSPA6, HSP90 and HSP 70 have recently been reported to be downregulated in good quality buffalo sperm (Binsila et al., 2021). Interestingly, the interaction of HSPA2 with BAG6 (BCL2-associated athanogene 6; formerly BAT3, HLA-B-associated transcript 3) has been demonstrated to regulate its stability thereby making BAG6 a molecular target for idiopathic male infertility (Sasaki et al., 2008; Bromfield et al., 2015). Although considered a nuclear protein, we identified BAG6 (along with HSPA2) amongst the sperm-surface proteins. The evidence regarding subcellular localization of HSPA2 is inconclusive. Some studies have reported its intracellular presence in the spermatozoa (Redgrove et al., 2012; Redgrove et al., 2013) whereas others reported it on the sperm surface (Naaby-Hansen and Herr, 2010; Motiei et al., 2013). Further studies are, however, warranted to determine and validate the predicted subcellular localization of HSPs and other intracellular proteins identified in this study.

## 5 Conclusion

Different protein combinations are responsible for sperm survival, function and preservation and the associated process appears to be species-specific. A shotgun proteomics-based deep proteomic profile of the buffalo sperm surface was generated in this study and >1,600 distinct protein isoforms were reported on the sperm surface. These proteins are implicated in the regulation of male fertility by affecting crucial processes such as spermatogenesis, maturation, protection (immunomodulation), capacitation and zona-binding or penetration. The buffalo sperm-surface proteome fingerprint generated in this study can help to decipher the functional role of these proteins in the acquisition of fertilizing ability by the buffalo sperm. Besides, since the samples used in our study were taken from fertile bulls of proven fertility, it would be interesting to compare the pattern of abundance of sperm surface protein with low fertile males. Therefore, this study is a welcome step to obtaining a greater understanding of sperm surface that is fundamental for the survival and performance of mammalian sperm.

## Data Availability

The LC-MS/MS generated data are available via ProteomeXchange (https://www.proteomexchange.org/) with the identifier PXD028026. Further inquiries can be directed to the corresponding author.

## Abbreviations

BD/DEFB, Beta-defensin; BuBD-129, Buffalo beta-defensin-129; GPI, Glycosylphosphatidylinositol; FRT, Female reproductive tract; MRT, Male reproductive tract; IF, Immunofluorescence; KOBAS, KEGG orthology-based annotation system; LC-MS/MS, Tandem Mass Spectrometry; PI-PLC, Phosphoinositide-phospholipase C.
