# Supplementary figures and images for: The proteomic landscape of sperm surface deciphers its maturational and functional aspects in buffalo
Source: Front Physiol. 2024 Jun 28;15:1413817. doi: 10.3389/fphys.2024.1413817 (PMC11239549; doi:10.3389/fphys.2024.1413817)

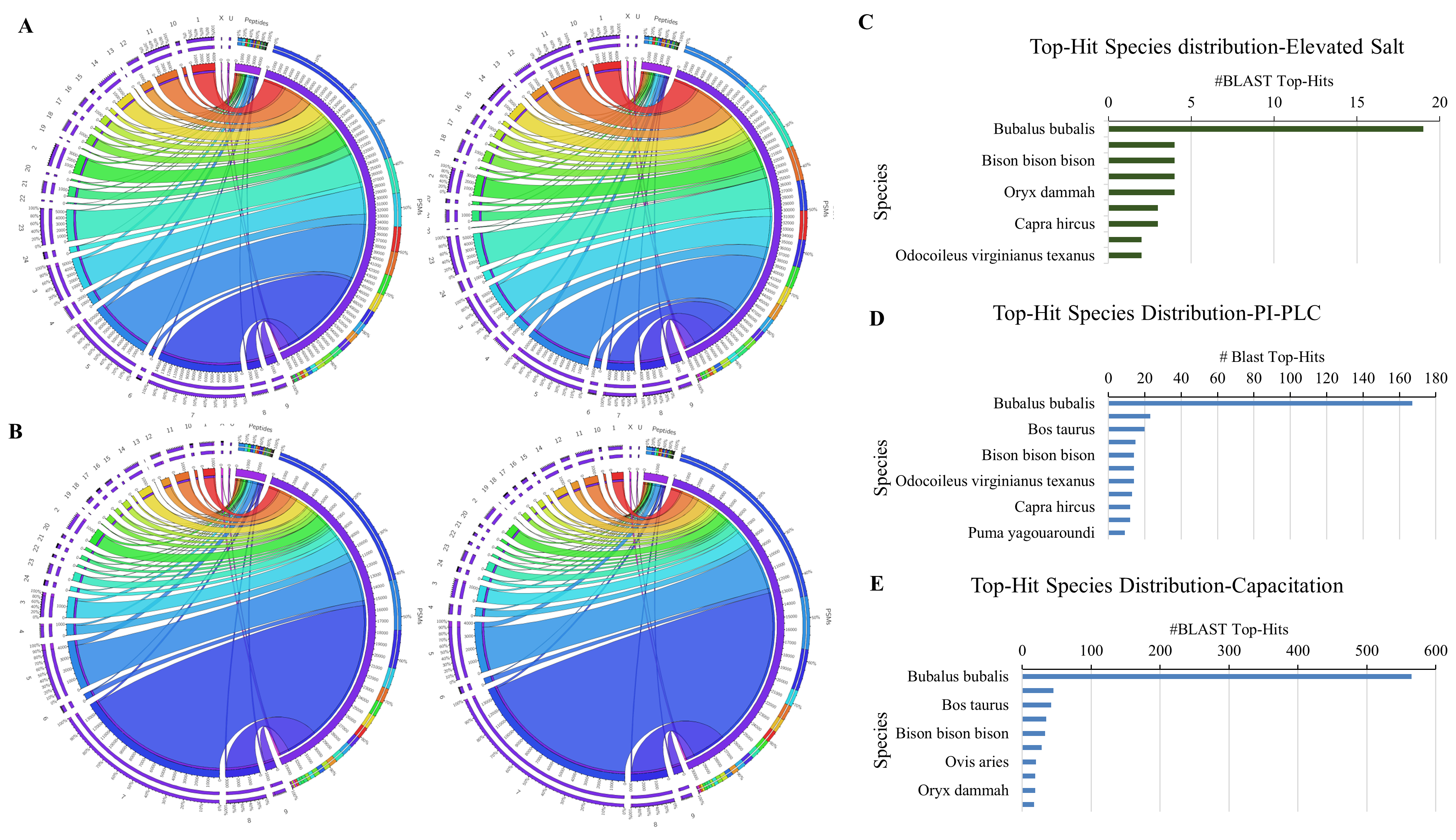

Supplement: Supplementary file 1 [file Image3.TIF]

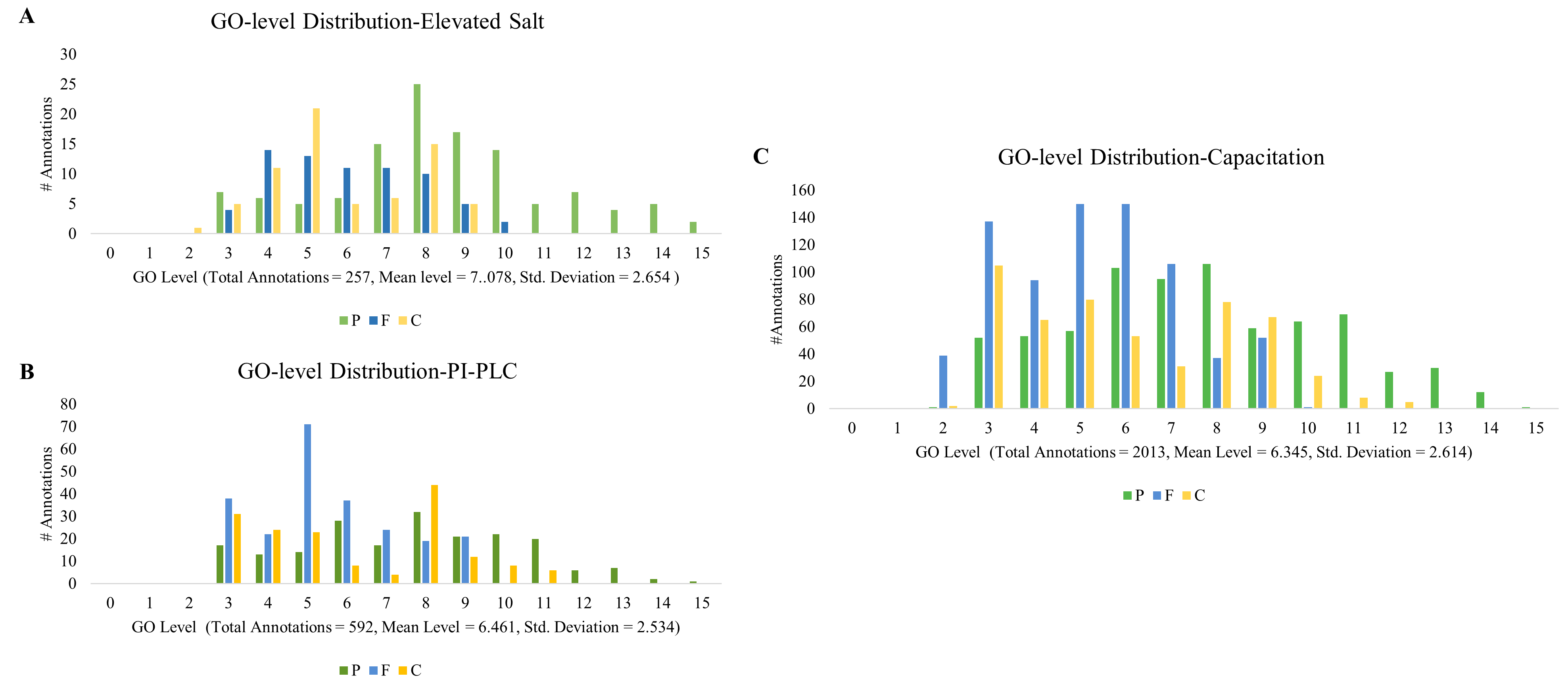

Supplement: Supplementary file 2 [file Image4.TIF]

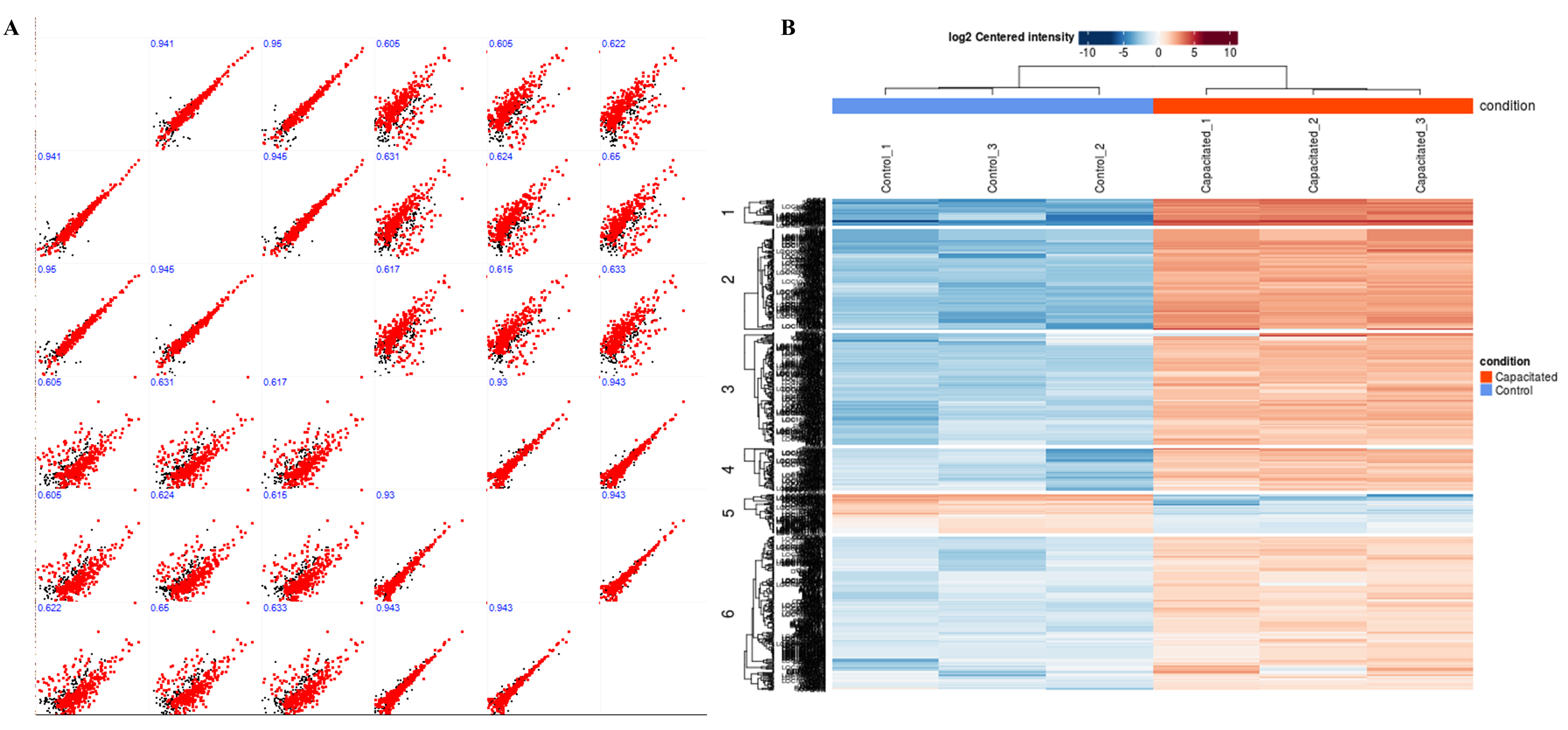

Supplement: Supplementary file 3 [file Image2.TIF]

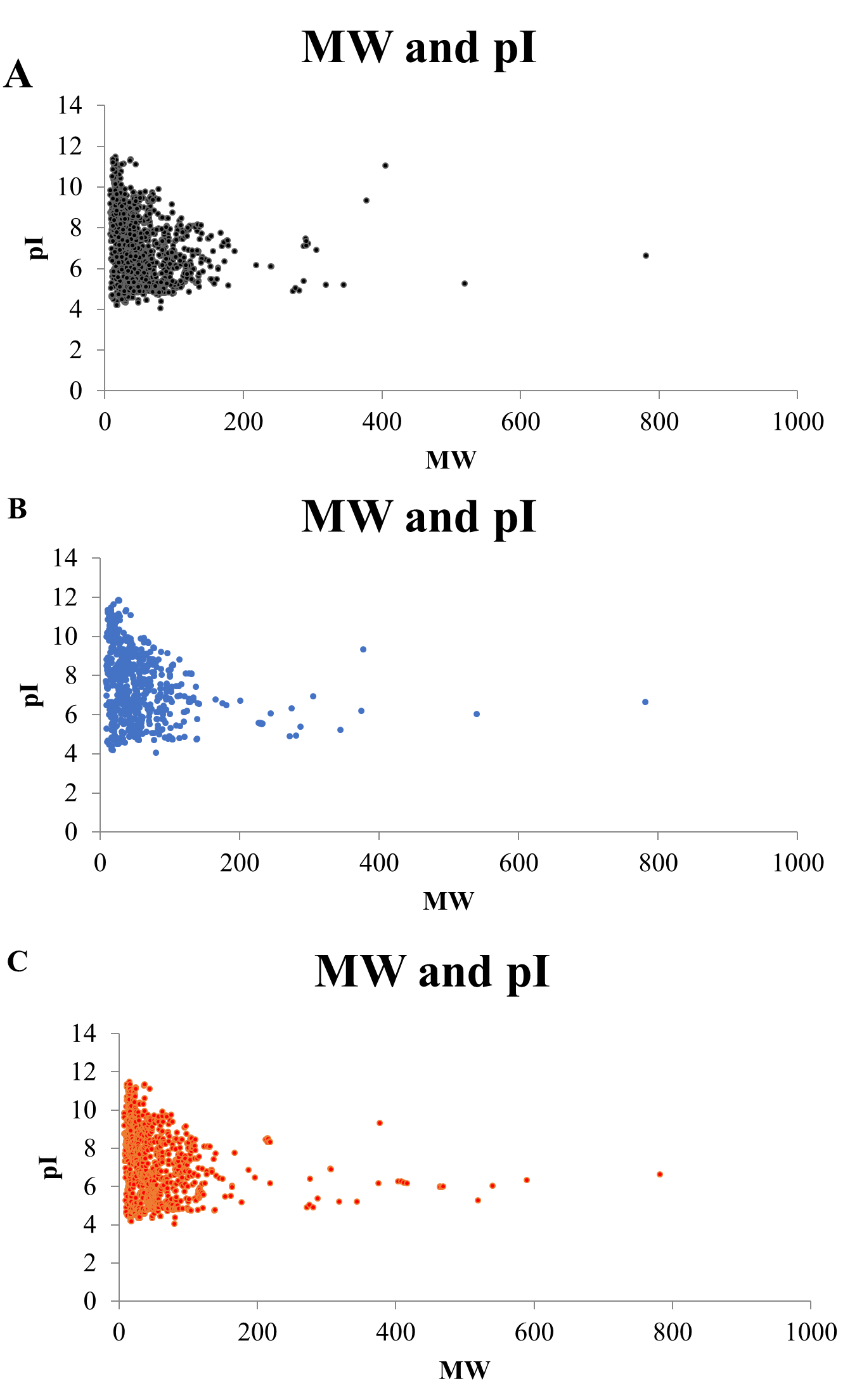

Supplement: Supplementary file 4 [file Image1.TIF]
